# Trachyonychia Secondary to Acitretin Usage

**DOI:** 10.7759/cureus.6703

**Published:** 2020-01-19

**Authors:** Taylor Thieman, Dillon Clarey, Richard A Johnson, Ryan M Trowbridge

**Affiliations:** 1 Dermatology, University of Nebraska Medical Center, Omaha, USA; 2 Dermatology, Massachusetts General Hospital, Boston, USA

**Keywords:** trachyonychia, acitretin, retinoids

## Abstract

Trachyonychia is a disease of the nail matrix that most commonly presents with sandpaper-like roughness of the nails. Retinoids are known to cause several nail abnormalities, likely due to their anti-proliferative effects. Despite this, no cases have been previously reported on the association of acitretin (second-generation retinoid) with trachyonychia. We present a single case of trachyonychia associated with acitretin that subsided following medication cessation.

## Introduction

Trachyonychia (“rough nails”) is a disorder of the nail matrix. It is most often a clinical diagnosis, although a nail biopsy (reserved for severe or recalcitrant cases) may provide insight into the underlying cause [1]. Despite its characteristic appearance, trachyonychia can present similarly to other nail unit diseases, such as onychomycosis and onychorrhexis (shares in the longitudinal ridging but lacks sandpaper-like roughness) [2,3]. Thus, maintaining a broad differential is important [4].

The cause of trachyonychia is most often idiopathic. Dermatologic conditions such as alopecia areata, lichen planus, ichthyosis vulgaris, and psoriasis have been implicated [2,5]. Additionally, chemotherapeutics, such as vincristine, and kinase inhibitors, such as imatinib mesylate, have been reported to cause trachyonychia [6,7]. Retinoids cause several different nail abnormalities due to their anti-keratotic effects. In this report, we describe a case of trachyonychia that began after two months usage of daily acitretin 25 mg in a patient being treated for palmoplantar keratoderma and psoriasis. This is the first reported association of acitretin (second-generation retinoid) with trachyonychia.

## Case presentation

A 65-year-old Indian male with a past medical history of undifferentiated arthritis presented to the dermatology clinic for palmoplantar keratoderma. On physical examination, there were thick, yellow, callous-like sheets of hyperkeratotic skin on the palms and soles. On the extensor elbows, pink plaques with silvery scale were present. There were no nail findings noted. Based on these examination findings, a diagnosis of plaque psoriasis was made.

After a thorough discussion of management options, acitretin 25 mg daily combined with hand-foot narrow band UVB phototherapy was selected as the treatment modality (his inflammatory arthritis had been quiescent for years). The patient was counseled to limit his alcohol consumption while taking acitretin.

**Figure 1:**
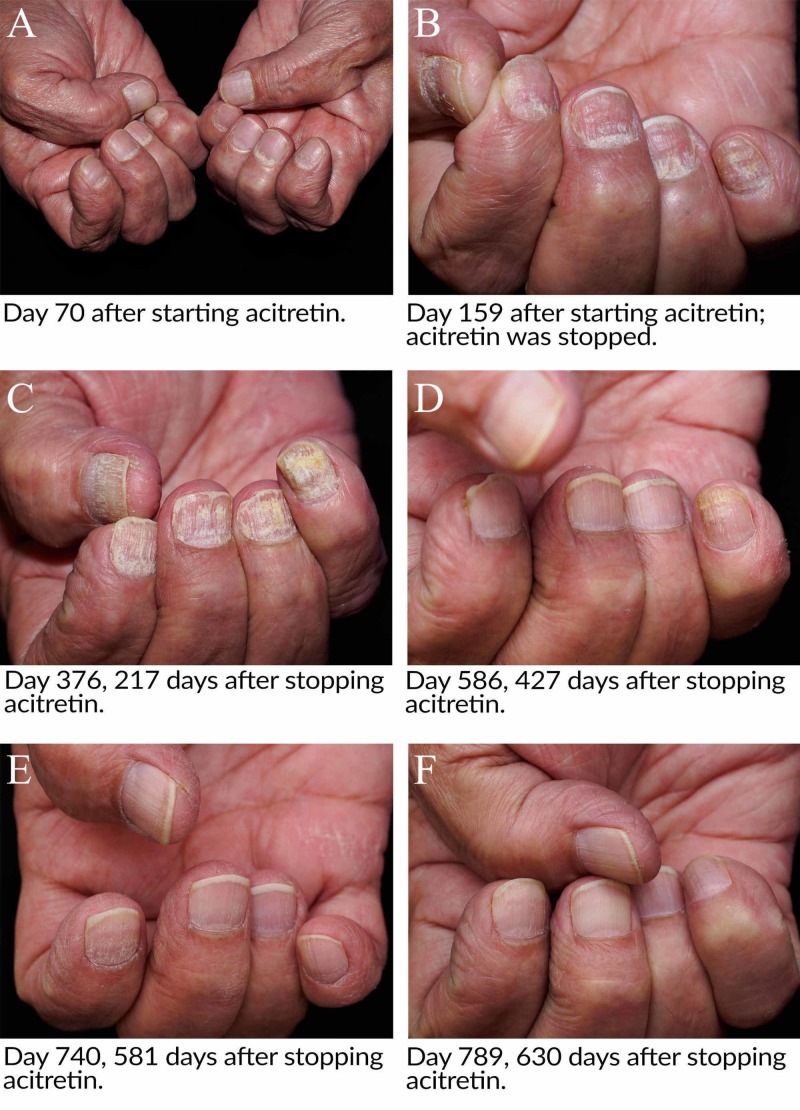
The progression of rough trachyonychia of the bilateral hands after starting and stopping (day 159) acitretin.

Approximately two months after starting acitretin, coarse sandpaper-like changes were noted at the proximal 1-2 mm of the nail plate of all 20 nails (Figure 1A).

As the nails continued to grow out over the upcoming months, these changes continued to affect the nails. Approximately five months after starting acitretin, it was stopped due to nail discomfort experienced by the patient (Figure 1B). The patient resumed alcohol intake at that time. 

Roughly seven months after discontinuing acitretin, the patient’s nails continued to worsen, now with the involvement of the proximal and distal nail plates by coarse fissuring and roughness (Figure 1C). Over the subsequent seven months, the nails experienced significant improvement (Figure 1D) that was seen at a follow-up at roughly 19 months (Figure 1E). By approximately 21 months after stopping acitretin, they were back to baseline (Figure 1F). The patient’s toenails followed a similar, although more protracted, course. They at first worsened to peak dystrophy about 19 months after acitretin discontinuation. At 21 months after discontinuation, they showed significant, although incomplete, improvement.

## Discussion

Trachyonychia is a disorder of the nail matrix that has two variants: opaque (most common) and shiny. Opaque trachyonychia is characterized by excessive longitudinal ridging of the nail that results in a sandpaper-like roughness. The less common shiny variant of trachyonychia also results in multiple pits in addition to excessive longitudinal ridging, but the nails retain their natural shine. As some cases involve all nails of the hands and feet (more often seen in children), this condition has also been termed “twenty nail dystrophy.” However, the involvement of all 20 nails is not requisite for diagnosis, and the severity from nail to nail may differ [5]. 

Acitretin is a second-generation retinoid that alters gene transcription by binding to retinoic acid receptors and retinoid X receptors within the nucleus of cells [6]. This gene expression modulation leads to normalization of epidermal growth and keratinization, allowing the retinoid to serve both anti-proliferative and anti-neoplastic properties [7]. Although this mechanism is beneficial in treating many dermatologic conditions typified by excessive keratinization (psoriasis, acne, keratodermas), it may also lead to unwanted side effects [7]. Side effects associated with retinoid usage include several mucocutaneous (rhinitis, cheilitis, xerosis) and nail growth disorders (nail thinning, softening, increased fragility), onychoschizia (splitting of the distal nail), Beau’s lines (horizontal depressions resulting from a disruption in proximal nail matrix function most often arising several weeks after an illness), subungual hemorrhage, transverse leukonychia (white bands), and paronychia [6,8-11]. Isotretinoin, a first-generation retinoid, has been associated with median nail dystrophy (longitudinal split in center of nail with transverse cracks projecting laterally), elkonyxis (nail defect that appears punched out near the lunula), bilateral onycholysis (nail plate separation from the underlying distal nail bed), and pyogenic granulomas [12].

Paradoxical to our case, retinoids have been described as possible treatment options for patients with idiopathic recurrent trachyonychia and occupational trachyonychia in several studies [13-15]. The first was a case report of a patient with a past history of psoriasis who worked as a jeweler and subsequently developed occupational trachyonychia secondary to a microtrauma; this was successfully treated with 0.3 mg/kg of acitretin daily [13]. The second publication was a case series that discussed the usage of alitretinoin (first-generation retinoid) 30 mg daily for at least three months in the management of idiopathic recurrent trachyonychia in 21 patients. This study reported some improvement in 98.1% of 210 nails and complete improvement in 22.9% of 210 nails after three months of alitretinoin treatment [14]. Lastly, acitretin 0.4 mg/kg (25 mg) in combination with topical clobetasol 8% was successfully used to manage idiopathic trachyonychia with nail resolution after roughly one year [15].

Despite these reported management successes, it is important to note that the treatment of trachyonychia is primarily for cosmetic reasons as well as quality-of-life issues as spontaneous resolution over the span of several years is often seen [2,16]. Resolution in children is typically more rapid than in adults (median of 32.5 months versus 77.0 months, respectively, p = 0.0002), possibly secondary to a decrease in the rate of linear nail growth with age [2]. Thus, more conservative options, including mild emollients and nail polish, are typically utilized first in patients who do desire treatment. If these do not improve appearance, topical medications (calcipotriol/betamethasone ointment, psoralen plus UVA, or triamcinolone injections) have shown management success [17]. Less commonly used options include a multitude of systemic medications (biotin, cyclosporine, corticosteroids) [18]. 

The above case did show worsening of nail disease after stopping acitretin. Acitretin is cleared through the biliary tract or renally within one month after drug cessation. However, acitretin is re-esterified to etretinate with concurrent alcohol usage [19]. Etretinate is lipophilic and has a half-life of 80-160 days [20]. This patient did resume alcohol intake at the time of acitretin stoppage; thus, we hypothesize that etretinate formation and toxicity may have been the causes of prolonged nail improvement. 

The anti-keratotic properties of retinoids are useful for the treatment of several dermatologic conditions and may be used for conditions of abnormal keratinization of the nail plate. However, the demonstration here that acitretin appears to be capable of inducing trachyonychia is important to recognize. Our patient had normal fingernails prior to acitretin treatment and again 21 months after cessation of acitretin. The slower resolution of the toenails is expected given their slower rate of growth. It is unclear if a patient already experiencing nail disease could develop worsening or new nail changes during treatment with acitretin, but reasonable to consider if observed during the treatment course. The mechanism of these changes is unknown, but our hypotheses include altered binding of retinoid receptors known to be present in the nail matrix or altered downstream effects from retinoid receptor binding. 

## Conclusions

Although retinoids have shown utility in the management of trachyonychia, a paradoxical reaction is also possible. Trachyonychia is a rare side effect of acitretin usage.
